# Molecular Spectrum Capture by Tuning the Chemical Potential of Graphene

**DOI:** 10.3390/s16060773

**Published:** 2016-05-27

**Authors:** Yue Cheng, Jingjing Yang, Qiannan Lu, Hao Tang, Ming Huang

**Affiliations:** 1 Wireless Innovation Lab of Yunnan University, School of Information Science and Engineering, Kunming 650091, Yunnan, China; chengyue@ynu.edu.cn (Y.C.); luqiannan@ynetc.gov.cn (Q.L.); 2 Radio Monitoring Center of Yunnan Province, Kunming 650228, Yunnan, China; tangh@ynrm.org.cn

**Keywords:** graphene, surface plasmons, sensors, molecules

## Abstract

Due to its adjustable electronic properties and effective excitation of surface plasmons in the infrared and terahertz frequency range, research on graphene has attracted a great deal of attention. Here, we demonstrate that plasmon modes in graphene-coated dielectric nanowire (GNW) waveguides can be excited by a monolayer graphene ribbon. What is more the transverse resonant frequency spectrum of the GNW can be flexibly tuned by adjusting the chemical potential of graphene, and amplitude of the resonance peak varies linearly with the imaginary part of the analyte permittivity. As a consequence, the GNW works as a probe for capturing the molecular spectrum. Broadband sensing of toluene, ethanol and sulfurous anhydride thin layers is demonstrated by calculating the changes in spectral intensity of the propagating mode and the results show that the intensity spectra correspond exactly to the infrared spectra of these molecules. This may open an effective avenue to design sensors for detecting nanometric-size molecules in the terahertz and infrared regimes.

## 1. Introduction

Surface plasmon polaritons (SPPs), which are electromagnetic excitations propagating along the interface between a dielectric and a metal [1], offer a promising solution to control electromagnetic waves at subwavelength scale. These electromagnetic surface waves arise via the coupling of the electromagnetic fields to oscillations of the conductor’s electron plasma [2]. Owing to their advantages of novel optical properties, such as selective absorption and scattering of light, subwavelength electromagnetic confinement and local optical field enhancement, SPPs have attracted a great deal of attention and have been widely used in integrated optics, chemosensing, biosensing, *etc*. [3,4,5,6,7,8,9]. Among the available plasmonic materials, the frequency range of which lies in the visible to near-infrared, noble metals are usually chosen to support SPPs for their relatively low losses [10]. However, in the mid-infrared to terahertz frequency, due to the difficulty in controlling and varying the permittivity functions of noble metals and the existence of material losses, only loosely bound surface waves can be supported by noble metals. Recently, experiments confirmed that doped graphene supports surface plasmons as well [11]. Graphene, a newly two-dimensional material with a thickness of only one atom, is considered a novel plasmonic material for the terahertz to infrared spectral region [12,13,14]. Graphene surface plasmons (GSPs) exhibit exceptional electrical tunability, low intrinsic loss, highly confined optical field and other fascinating properties due to the fact graphene has special electronic energy band structures [11,15,16,17,18]. Various graphene nanostructures were proposed to support GSPs modes. Currently, the design of optical devices using graphene has become a hot research topic. A variety of devices such as nanoribbons [19,20], nano-discs [21], graphene-metal plasmonic antennas [22] and graphene resonance sensors [23] have been proposed. Moreover, several experiments [10,24,25,26,27] have also indicated that a graphene layer can tightly coat a dielectric nanowire due to van der Waals force. Graphene-microfiber/nanowire combined system has been proposed for various applications [24,25,26,27,28,29]. Detection and sensing at the molecular level have attracted much attention and some graphene-based schemes were presented [23,30]. Graphene-based platforms for the detection of individual gas molecules [31], protein monolayers [32] and DNA [33] open exciting prospects for chemo- and bio-sensing. In addition, an ultra-compact tunable plasmon ring resonator which is composed of a monolayer of graphene and a graphene ring was proposed and analyzed numerically by Huang *et al*. [34]. The characteristics of the plasmon ring resonator, such as resonance frequency tenability and high quality factor were analyzed numerically. However, the application of the resonator as a sensor has not been explored yet.

In this study, we investigate the sensing characteristic of a tunable graphene-coated dielectric nanowire (GNW) waveguide, which could be used to detect molecules. The transverse resonant modes are simulated numerically in detail using the finite element method, and validated by theoretical analysis. It is found that a small change in the chemical potential can induce the resonant mode to shift by a large frequency range. We also show that the mode behavior of the GNW can be influenced by the relative permittivity of a thin layer dielectric which is supposed to be the analyte attached to the outer side of GNW waveguide. Remarkably, the GNW is proven to play a pivotal role as a highly sensitive platform for broadband molecular sensing.

## 2. Physical Model and Principles

**Figure 1:**
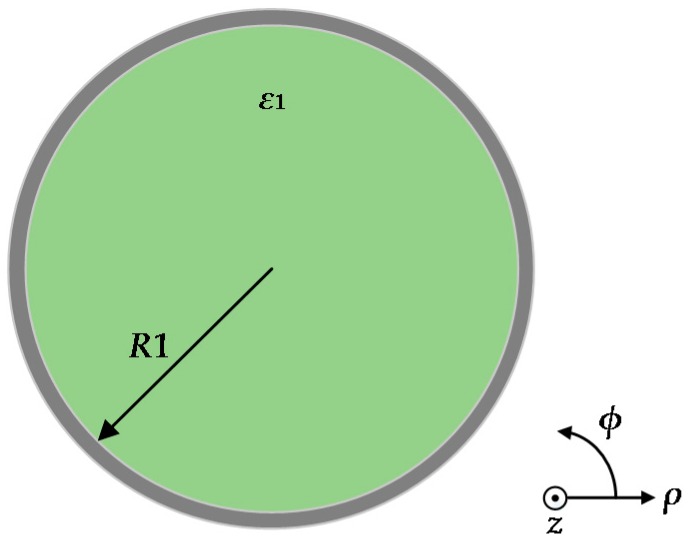
Schematic illustration of the graphene-coated dielectric nanowire waveguide.

The GNW is a graphene-coated dielectric nanowire which is excited by a graphene ribbon. The geometry of the GNW is shown in Figure 1. A dielectric nanowire core of relative permittivity ε_1_ = 2.1ε_0_ is surrounded by a graphene layer with a conductivity σ*_g_* expressed by the Kubo formula [35,36], which represents a finite temperature as σ*_g_*= σ_intra_ + σ_inter_ with the intraband and interband contributions being:

(1) $$\sigma_{\mathrm{intra}}=\frac{2ie^{2}T}{\hbar^{2}\pi \left(\omega +i\Gamma \right)}\ln\left[2\;\cosh\left(\frac{\mu_{c}}{2T}\right)\right]$$

(2) $$\sigma_{\text{inter}}=\frac{e^{2}}{4\hbar^{2}}\left[\frac{1}{2}+\frac{1}{\pi }\text{arctan}\left(\frac{\hbar \omega -2\mu_{c}}{2T}\right)-\frac{i}{2\pi }\ln\frac{{\left(\hbar \omega +2\mu_{c}\right)}^{2}}{{\left(\hbar \omega -2\mu_{c}\right)}^{2}+{\left(2T\right)}^{2}}\right]$$

where ε_0_ is the permittivity of vacuum, *T* = 300 × *k_B_* is the temperature energy, *k_B_* is the Boltzmann constant, μ*_c_* is the chemical potential, Γ represents the carrier scattering rate and *ħ* denotes the reduced Planck constant. The radius of the dielectric nanowire is R1, the thickness of the graphene is 0.5 nm as an anisotropic layer. The permittivity of the graphene is approximated by ε = 1 + iσ*_g_*/ε_0_ω*t* as proposed in [37], where *t* is the effective thickness. The longitudinal component of electric field E*_z_* and magnetic field Hz could be represented as $E_{z}^{\left(1\right)}=A_{m}I_{m}\left(\mu_{1}\rho \right)\exp\left(jm\phi \right)\exp\left(j\beta z\right),\;H_{z}^{\left(1\right)}=B_{m}I_{m}\left(\mu_{1}\rho \right)\exp\left(jm\phi \right)\exp\left(j\beta z\right),$ for ρ < *R*, and $E_{z}^{\left(2\right)}=C_{m}K_{m}\left(\mu_{2}\rho \right)\exp\left(jm\phi \right)\exp\left(j\beta z\right),\;H_{z}^{\left(2\right)}=D_{m}K_{m}\left(\mu_{2}\rho \right)\exp\left(jm\phi \right)\exp\left(j\beta z\right),$ for ρ > *R*, where $\mu_{1}=\sqrt{\beta^{2}-\omega^{2}\varepsilon_{1}\mu_{0}}$ and $\mu_{2}=\sqrt{\beta^{2}-\omega^{2}\varepsilon_{2}\mu_{0}}$. *I_m_*(μ_1_*ρ*) and *K_m_*(μ_2_ρ) are the modified Bessel functions of the first and second kind (*m* = 0, 1, 2…). *A_m_*, *B_m_*, *C_m_* and *D_m_* are arbitrary constants which are determined by the boundary conditions. The terms μ_0_, *k*_0_ and ω are the magnetic permittivity, wavenumber in free space and radian frequency, respectively, μ_1_ and μ_2_ are related to the mode field confinement. By applying the relation between the longitudinal and transverse EM field components, and boundary condition, the eigen-equation for the m-th order mode [10] is:

(3) $$\left|\begin{array}{cccc} I_{m}\left(\mu_{1}R\right) & 0 & -K_{m}\left(\mu_{2}R\right) & 0\\ \frac{jm\beta }{-\mu_{1}^{2}R}I_{m}\left(\mu_{1}R\right) & \frac{\omega \mu_{0}}{\mu_{1}}\overset{´}{I_{m}}\left(\mu_{1}R\right) & \frac{jm\beta }{\mu_{2}^{2}R}K_{m}\left(\mu_{2}R\right) & -\frac{\omega \mu_{0}}{\mu_{2}}\overset{´}{K_{m}}\left(\mu_{2}R\right)\\ \frac{j\omega \varepsilon_{1}}{\mu_{1}}\overset{´}{I_{m}}\left(\mu_{1}R\right)-\sigma_{g}I_{m}\left(\mu_{1}R\right) & -\frac{m\beta }{\mu_{1}^{2}R}I_{m}\left(\mu_{1}R\right) & -\frac{j\omega \varepsilon_{2}}{\mu_{2}}\overset{´}{K_{m}}\left(\mu_{2}R\right) & \frac{m\beta }{\mu_{2}^{2}R}K_{m}\left(\mu_{2}R\right)\\ \sigma_{g}\frac{m\beta }{-\mu_{1}^{2}R}I_{m}\left(\mu_{1}R\right) & I_{m}\left(\mu_{1}R\right)-\sigma_{g}\frac{j\omega \mu_{0}}{\mu_{1}}\overset{´}{I_{m}}\left(\mu_{1}R\right) & 0 & -K_{m}\left(\mu_{2}R\right) \end{array}\right|=0$$

By solving Equation (3), we could get the propagation constant β of the m-th order mode and the corresponding EM field. The real part of β relates to the surface plasmons’ wavelength λ_SPs_ = 2π/Re(β), and the effective mode index is defined as Re(β)/*k*_0_. The imaginary part of β relates to the propagation loss of surface plasmons, and propagation length can be defined as 1/2Im(β), which means surface plasmons’ mode decays to 1/*e* of its original power after travelling such a distance. A larger μ_1_ (μ_2_) leads to *I_m_*(μ_1_ρ) [*K_m_*(μ_2_ρ)] increasing (decreasing) more quickly, which means the field is better confined near the graphene [10].

**Figure 2:**
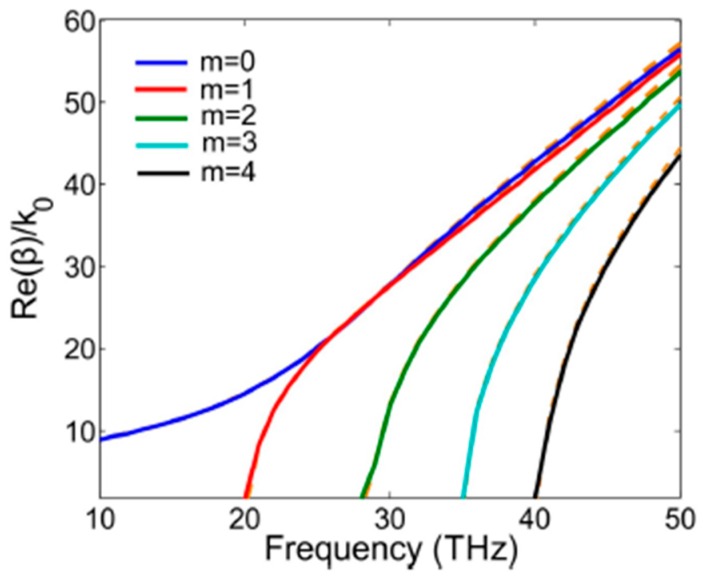
Theoretical (dotted lines) and simulation (solid lines) results for the dispersion relations of GSPs modes in the GNW.

Figure 2 shows the theoretical and simulation results for the dispersion relations of GSPs modes in GNW of the first five orders. The solid lines represent the simulation results while the dotted lines correspond to the theoretical results. We notice that *m* = 0 mode is cutoff-free and the effective mode indexes of all modes decrease monotonically with decreasing frequency. It also indicates a larger proportion of the mode energy resides inside the GNW near the graphene-nanowire at high frequencies, while at low frequencies, they are mainly localized outside the GNW.

## 3. Simulation and Analysis

### 3.1. Resonance Mode Analysis

**Figure 3:**
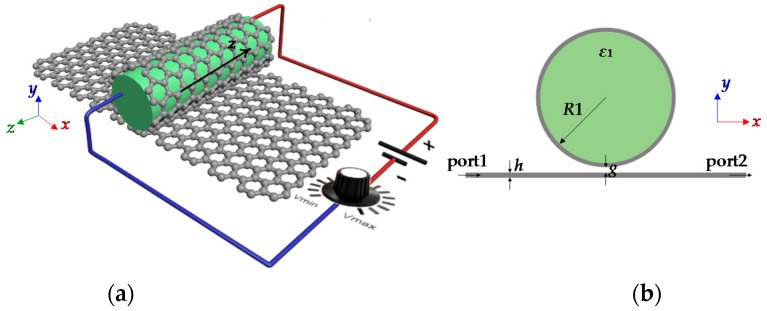
(**a**) Schematic diagram of the sensor model based on the cylindrical dielectric loaded with a thin layer graphene and monolayer graphene waveguide; (**b**) Cross-section of the structure.

The 3D geometrical structure of the GNW waveguide excited by a graphene ribbon is exhibited in Figure 3a. Figure 3b is a schematic cross-section of the structure. The GNW could be regarded as a graphene ribbon rolled around a dielectric nanowire, which can be silicon and GaN. In this paper we choose a dielectric nanowire with a relative permittivity ε_1_ = 2.1ε_0_ and magnetic conductivity μ_0_. The studied structure is embedded in air. The complex conductivity of the thin layer graphene colored in gray is given by the Kubo formula [35,36]. In this paper, we choose ambient temperature T = 300 *k*, chemical potential μ_c_ = 0.5 eV, and the carrier scattering rate Γ = 1.32 meV represents the carrier scattering rate.

**Figure 4:**
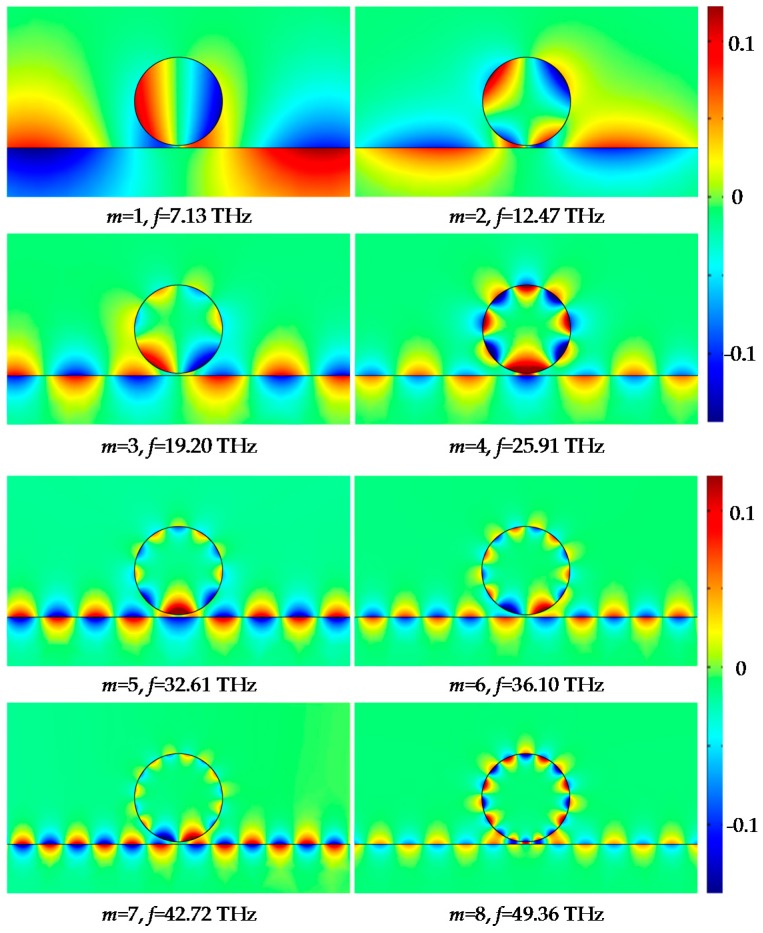
Simulation results of magnetic-field (H*_z_*) distribution at the resonant state of mode 1–8 for the GNW.

The transverse resonance characteristics of the GNW are studied based on the commercial finite element method (FEM) software COMSOL Multiphysics. In the simulation, the radius of the dielectric core is set to be *R*_1_ = 190 nm, the thickness of the graphene *h* = 0.5 nm, while the coupling distance between the graphene ribbon and the outer surface of the GNW is set to be *g* = 10 nm. The excitation source of unit amplitude, with magnetic vector parallel to the *z*-axis is set at port1 of the graphene ribbon. A radiation boundary condition is used to truncate the computational domain. The whole space is meshed by triangle grids except for the graphene ribbon and the graphene layer of the GNW, where smooth mapped mesh grids are adopted to ensure the simulation accuracy. The magnetic field distribution in the vicinity of the GNW is shown in Figure 4. Transverse resonant modes with orders of *m* = 1, 2, 3, 4, 5, 6, 7, 8 can be observed in the frequency range of 7.13 THz to 49.36 THz. It is seen that the magnetic fields are tightly confined to the surface of the GNW. Therefore, this area will be quite sensitive to the dielectric environment.

**Figure 5:**
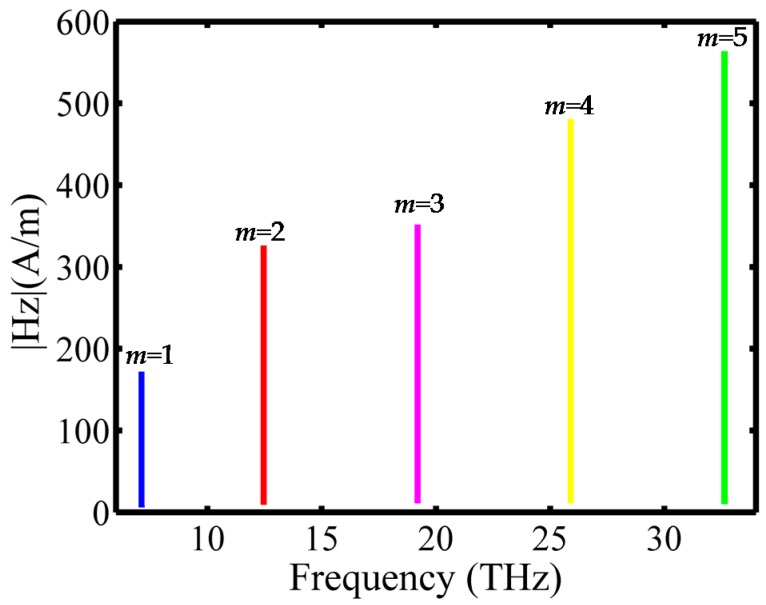
The resonant frequency spectrum.

In Figure 5, we plot the resonant spectrum of the GNW in the frequency of 7.0 THz–33.0 THz. It is simulated by frequency sweep. A probe is located at the point that possesses the maximum magnetic field in the resonant state to record the variation of |H*_z_*| with frequency. From left to right, the spectral lines correspond to modes 1, 2, 3, 4 and 5. The resonant frequency of the GNW is calculated and compared with the theoretical results, as shown in Table 1. A good agreement between the eigenfrequency (*f_e_*) and the resonant frequency (*f_r_*) confirms the effectiveness of the GNW.

### 3.2. The Dependence of the Resonant Spectrum on the Chemical Potential

**Figure 6:**
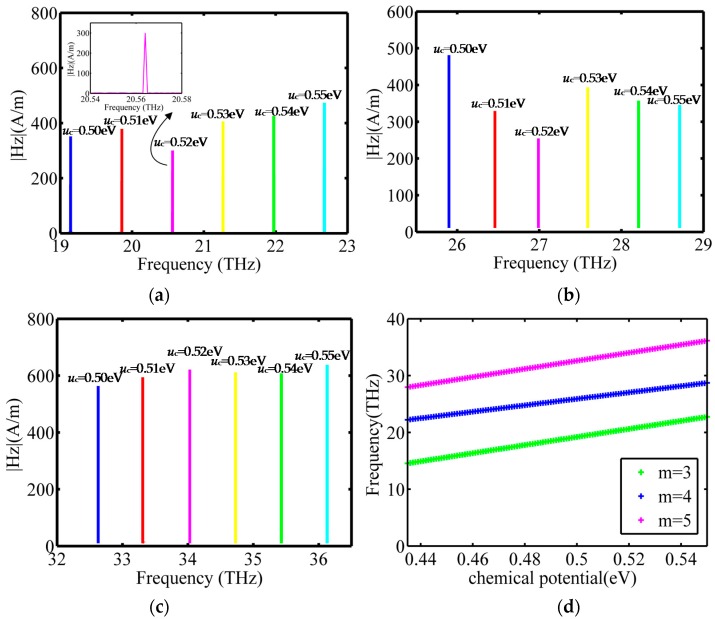
(**a**–**c**) Resonant frequency spectrum of modes 3, 4, 5 for a variation of chemical potential; (**d**) The relation between resonant frequency and the chemical potential.

In order to investigate the tunability of the resonant frequency of the GNW, frequency spectra with respect to the change of the chemical potential of graphene are simulated. The chemical potential in graphene can be controlled with potentially fast speed using a FET structure [11], where the approximate relationship is μ_c_ ∝ (*V*_g_)^1/2^ and *V*_g_ is the gate voltage [38]. It has been shown that a chemical potential change of 0.1 eV can be obtained with a gate voltage of a few volts [11,39], so we applied a varied gate voltage to control the chemical potential of the graphene. We suppose that the chemical potential of graphene varies from 0.5 eV to 0.55 eV with intervals of 0.1 eV, and then we extract the variation of magnetic field amplitude with frequency. Taking modes 3, 4, 5 as an example, the simulation results are shown in Figure 6a–c. It is seen that the spectra are red shifted as the chemical potential of graphene increases. It is clear that the response to an increase of 0.1 eV in the chemical potential of graphene is a frequency shift of 707.2, 562.0, and 707.2 GHz on average. The inset portrays the amplification of the resonant peaks in the frequency range of 20.54–20.58 THz when the chemical potential is 0.52 eV. The resonant frequency varies linearly with chemical potential, as shown in Figure 6b.

## 4. Sensing Application

### 4.1. Sensing Characteristics

**Figure 7:**
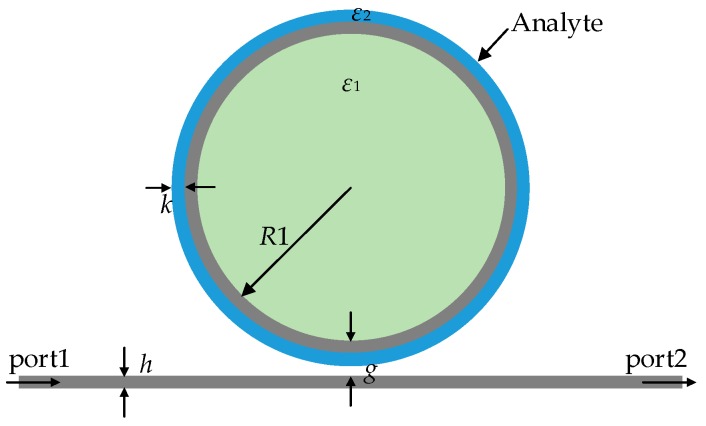
A schematic illustration of the GNW sensor model with a layer of analyte absorbed to the surface of graphene.

**Figure 8:**
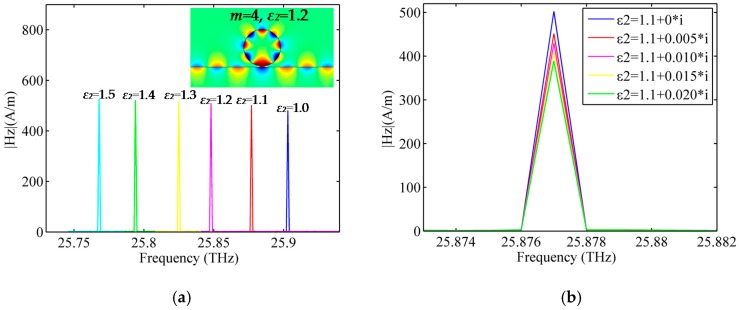
The resonant frequency spectra of mode 4 for a variation of the real part (**a**) and imaginary part (**b**) of analyte permittivity. The inset denotes the magnetic-field (H*_z_*) distribution at the resonant state when ***ε****_2_* = 1.2.

To explore the sensing properties of the GNW, we suppose that a layer of analyte with thickness (*k*) of 8 nm is attached to the surface of the graphene, as shown in Figure 7. Relative permittivity of the attached analyte is denoted as *ε*_2_, and the surrounding medium is air. In order to investigate the sensing performance of the GNW, resonant frequency spectra with respect to the change of analyte permittivity were simulated. Firstly, we suppose that the analyte is lossless and the real part of the relative permittivity varies from 1.0 to 1.5 with an interval of 0.1. Taking mode 4 as an example, simulation results are shown in Figure 8a. The inset denotes the magnetic-field (Hz) distribution at the resonant state of mode 4 when the analyte is absorbed to the surface of the GNW. It is seen that the spectra are red shifted with increasing permittivity. The relative frequency shift in response to an increase of 0.1 in analyte permittivity is a significant resonant frequency downshift of 27.4 GHz on average. Then, we investigate the impact of the imaginary part of the analyte on the resonant spectra. For a variation of imaginary part, simulation results of the resonant frequency spectra are depicted in Figure 8b. It is clear the imaginary part of the analyte permittivity mainly influences the amplitude of the spectra.

### 4.2. Molecular Sensing

**Figure 9:**
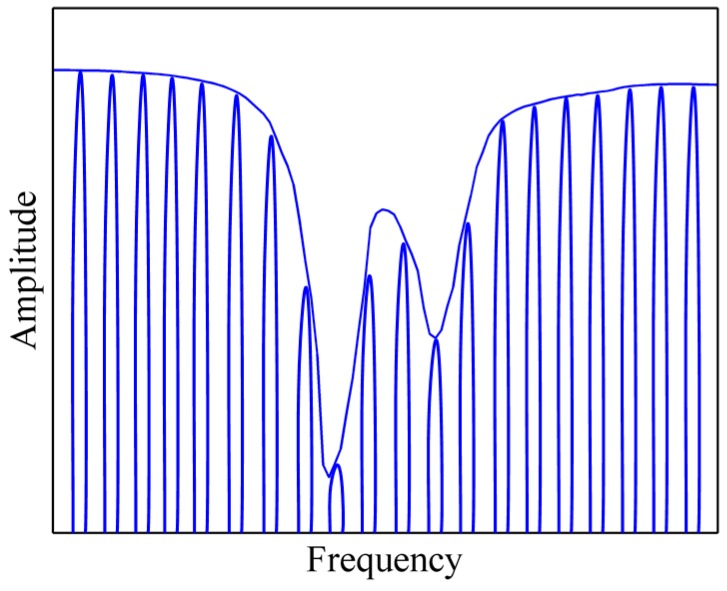
A schematic illustration of molecular spectrum capture.

It is known that unambiguous identification of low concentration chemical mixtures can be performed by broadband enhanced infrared absorption (BEIRA) [30], due to the infrared spectrum of the molecule which acts as a unique fingerprint. In the mid-infrared to terahertz regime, graphene sheet is an ideal carrier of highly confined surface plasmon modes. Besides, from the preceeding analysis we obtained that the resonant frequency of the GNW sensor can be flexibly tuned by adjusting the chemical potential of graphene, and amplitude of the resonance peak varies linearly with the imaginary part of the analyte permittivity, so we predicted that the resonance peak can act as a probe for capturing the molecular spectrum which may be reflected in the change of resonance peak amplitude, as shown in Figure 9.

In what follows, we perform mode analysis to demonstrate the capture of molecular spectrum with the GNW. The absorption spectrum of the analyte used in the simulation is obtained from the National Institute of Standards and Technology (NIST, Gaithersburg, MD, USA) [40]. The intensity of the surface plasmon along the *z* direction is *I = e^−z/L^*. Here, *z* denotes the length of the GNW which is put along the z axis as shown in Figure 7. L is the propagation length of the mode, which is defined as *L*/λ_0_ = 1/Im(*n_eff_*). λ_0_ is the free space wavelength, *n_eff_* is the effective mode index. We set *I_mol_* and *I*_0_ to indicate the intensities with and without molecules deposited on the graphene surface. 

**Figure 10:**
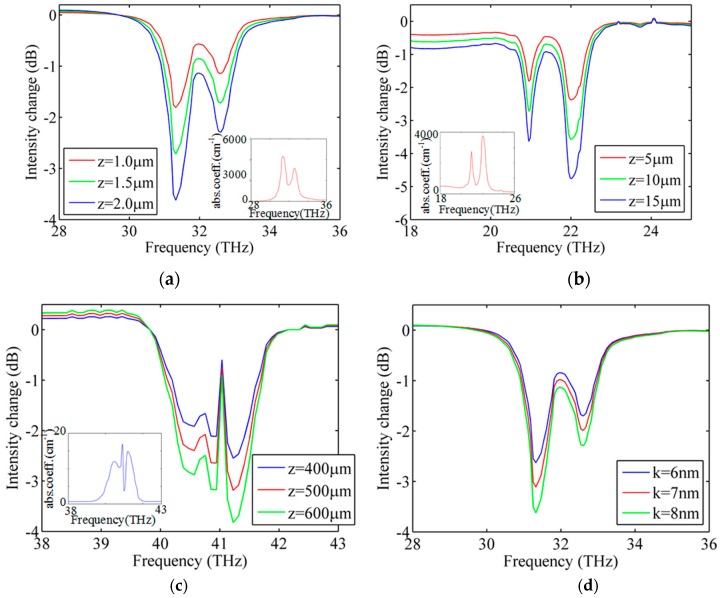
Intensity change caused by presence of analyte. (**a**) Ethanol; (**b**) Toluene; (**c**) Sulfurous anhydride; (**d**) Ethanol in different thickness. In panels “**a**–**c**”, the insets show the absorption spectrum of the analyte in the same frequency region as the main axis.

Taking the liquid ethanol molecule as an example, the normalized intensity spectra (*I_mol_*/*I*_0_) are illustrated in Figure 10a. It is seen that the variation of the signal directly maps the strong characteristic features of the molecule infrared absorption spectrum shown in the insect of Figure 10a. More importantly, a sample only 2.0 μm long can produce a gigantic 3 dB (50%) drop in intensity with the proposed sensor, which indicates exceptional sensitivity. 

To achieve such a wide band detection, the surface plasmon modes should have two features, *i.e.*, confinement to the surface of plasmonic materials and existing in an extended frequency band, within which the molecular vibrational modes appear. To demonstrate the tunability features of the SPP mode, liquid toluene molecules of which the characteristic absorption spectrum is localized in the frequency range of 18–24 THz is adopted in the simulation. Similarly, an intensity change which directly maps the fingerprint of the molecule is obtained, as shown in Figure 10b. But since the absorption loss of toluene molecule is smaller than that of ethanol, it needs a 20 μm long sample along the *z* direction to induced a 3 dB change in mode intensity. Figure 10c shows the simulation results of the gaseous sulfurous anhydride molecule, of which the characteristic absorption spectrum is localized in the frequency of 38–43 THz. As before, a transmission spectrum of the GNW sensor which maps well with the fingerprint of the molecule can also be obtained. Owing to the fact the absorption loss of a gas molecule is less than that of a liquid molecule, the sulfurous anhydride molecule needs a 600 μm long sample along *z* direction to induced 3 dB change in mode intensity. Taking again ethanol as an example, the variation of intensity spectra with respect to analyte thickness is shown in Figure 10d. It is clear that with the increase of the analyte thickness, the drop in intensity becomes deeper. Last but not least, the sensitivity can be improved considerably by changing the parameters of the GNW sensor, such as the radius, and the permittivity of the dielectric, which means that smaller scale samples can be detected. Therefore, it is possible to identify different chemical substances using the proposed sensor and it may provide an effective way for detecting nanometric-size molecules.

## 5. Conclusions

In summary, we demonstrate that plasmon modes of the GNW waveguide can be excited when coupled to a graphene ribbon. The GNW works as a probe for capturing a molecular spectrum. This is due to the factor that the resonant spectra of the GNW sensor can be flexibly tuned by adjusting the chemical potential of graphene, and the peak values of the spectra decrease linearly with the increase of analyte loss. Mode analysis reveals that intensity spectra of the GNW maps well with the absorption spectra of the molecules deposited on the surface of the graphene ring, which further verifies the broadband molecular sensing capabilities.

## Figures and Tables

**Table 1:** Comparison of the eigenfrequency (*f*_e_) and the simulated resonant frequency (*f*_r_) for modes 1 to 8 of the GNW.

Mode	1	2	3	4	5	6	7	8
*f*_e_(THz)	7.133	12.473	19.198	25.907	32.605	36.103	42.717	49.361
*f*_r_(THz)	7.131	12.469	19.189	25.893	32.590	36.093	42.712	49.357
